# Lipid overload meets S-palmitoylation: a metabolic signalling nexus driving cardiovascular and heart disease

**DOI:** 10.1186/s12964-025-02398-3

**Published:** 2025-09-02

**Authors:** Jingwen Zhang, Suying Wu, Yuantong Xu, Lei Zhang, Cong Cong, Menghe Zhang, Yonghao Jiang, Yang Liu

**Affiliations:** 1 Cardiovascular Department, Affiliated Hospital of Shandong University of Traditional Chinese Medicine, 42 Wenhuaxi Road, Jinan, Shandong China; 2 Foreign Language College, Shandong University of Traditional Chinese Medicine, No. 4655, Changqing University Science Park, Changqing District, Jinan, Shandong China; 3 Youth League Committee, Shandong Energy Xinwen Mining Group Staff college, No.81, Fengcheng East Street, Laiwu District, Jinan, Shandong China; 4 Traditional Chinese medicine department, The Second Hospital of Shandong University, Jinan, Shandong China; 5 Cardiovascular Department, The Second Affiliated Hospital of Shandong, University of Traditional Chinese Medicine, Jingba Road, Shizhong District, Jinan, 80 Shandong China

**Keywords:** S-palmitoylation, Lipid metabolism, Cardiovascular disease, Atherosclerosis, Heart disease

## Abstract

S-palmitoylation has emerged as a critical integrator of lipid overload and cardiovascular dysfunction. Disordered lipid metabolism inundates endothelial cells, vascular smooth muscle cells and macrophages with triglyceriderich lipoproteins, oxidized LDL and saturated fatty acids, expanding the intracellular palmitoylCoA pool and perturbing redox balance. Protein Spalmitoylation, the reversible attachment of palmitate to cysteine residues, converts excess palmitoylCoA into broad alterations in signalling and membrane dynamics. The FASN-ACSL-ZDHHC axis channel excess fatty acids into palmitoylCoA, which is transferred to pivotal proteins including the lipid transporter CD36, endothelial nitric oxide synthase (eNOS), key ion channels and the pyroptosis effector gasdermin D (GSDMD). Cycles of palmitate addition and removal regulate membrane residency, foam cell formation, nitric oxide production, calcium handling and inflammatory cell death, thereby linking lipid burden to atherosclerotic plaque growth, arrhythmogenic risk, heart failure progression and pulmonary hypertension. Therapeutic targeting of fatty acid uptake, palmitoyltransferases or thioesterases alleviates metabolic overload, restores endothelial reactivity and preserves myocardial viability in experimental models. This review synthesizes mechanistic and preclinical studies to delineate how lipiddriven protein palmitoylation reprograms cardiovascular physiology and pathology.

**Graphical Abstract:**

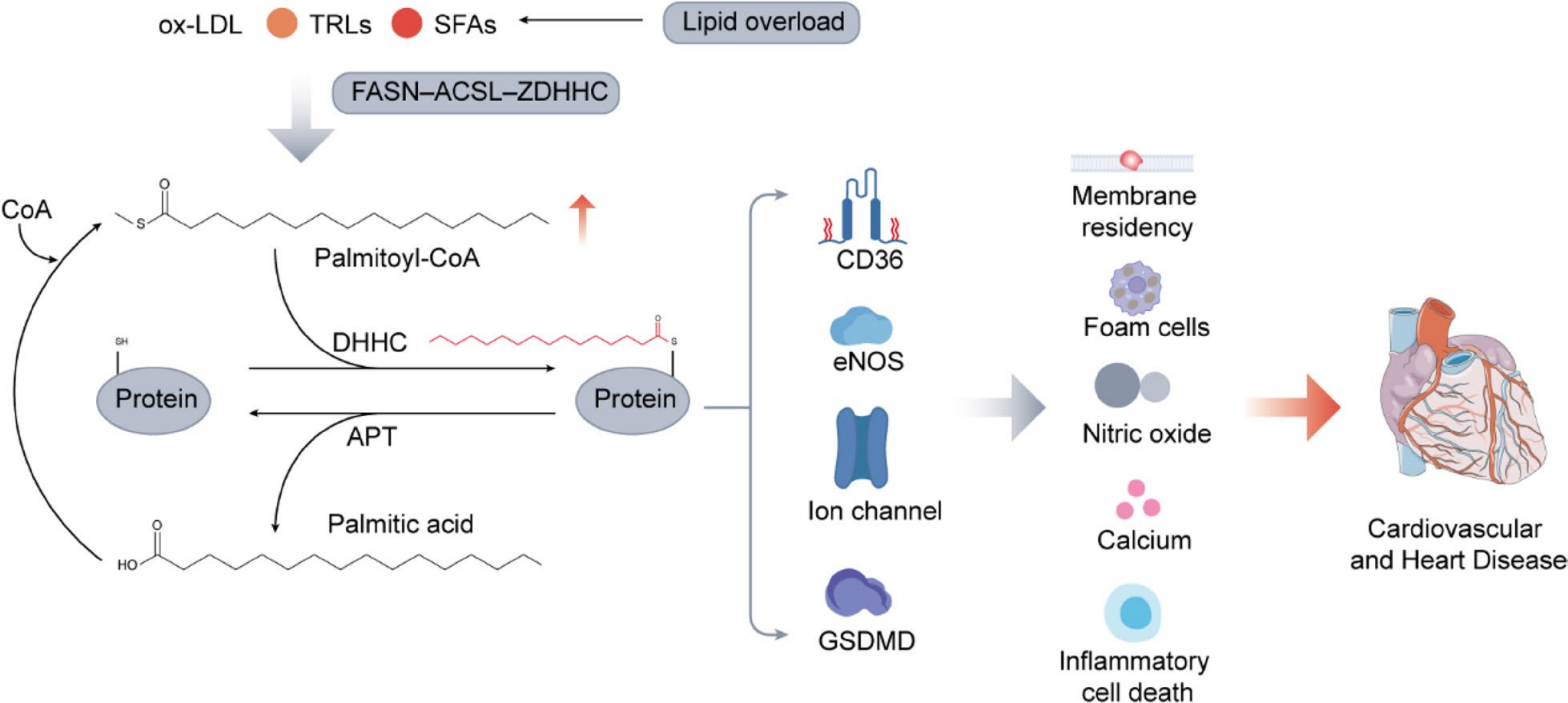

## Introduction

Cardiovascular disease (CVD) represents the leading cause of morbidity and mortality among non-communicable diseases worldwide, underscoring its critical importance for research. According to the 2021 Global Burden of Disease Study, the number of incident cardiovascular disease cases increased by 92.3%, from 34.74 million in 1990 to 66.81 million in 2021; over the same period, CVD‑related deaths rose by 57.5%, from 12.33 million to 19.42 million [1, 2]. Although high-income countries have achieved substantial reductions in age-standardized CVD mortality over the past decades through public health interventions and advanced medical technologies [3], Of the 20.5 million CVD‑related deaths in 2021, approximately 80% occurred in low‑ and middle‑income countries, reflecting a complex interplay of socioeconomic factors and metabolic disorders that continues to pose a formidable challenge for global public health governance. Among multiple risk factors, metabolic dysregulation—particularly lipid metabolism abnormalities—exhibits a strong association with CVD; insulin resistance, hypertension, and dyslipidemia constitute key components of metabolic syndrome and accelerate the development of atherosclerotic plaque formation and instability, thereby promoting severe cardiovascular events such as coronary heart disease and stroke [4].

Within the pathogenesis of CVD, lipid metabolism assumes a central role. Clinically, elevated triglyceride (TG) levels and low high-density lipoprotein cholesterol (HDL-C) are recognized as independent risk factors for atherosclerosis and associated cardiovascular events. A substantial body of evidence demonstrates that increased TG levels not only induce endothelial injury through the generation of TG-rich lipoprotein remnants—which trigger inflammatory cascades and macrophage uptake to promote foam cell formation and plaque development—but also alter hemorheological properties, exacerbating thrombogenic potential and significantly increasing the risk of myocardial infarction and stroke [5, 6]. Concurrently, reduced HDL-C impairs reverse cholesterol transport, leading to excess cholesterol accumulation in peripheral tissues—particularly the vascular endothelium—thereby promoting oxidative stress, endothelial dysfunction, and upregulation of adhesion molecules. These changes further intensify inflammatory responses and hemodynamic disturbances, creating a milieu conducive to plaque rupture and thrombus formation [7, 8]. Moreover, the strong association between non-alcoholic fatty liver disease (NAFLD) and dyslipidemia highlights the significance of hepatic metabolic derangements; dysregulated hepatic lipid and lipoprotein metabolism exacerbates cardiovascular risk through the liver–heart axis. Accordingly, management of lipid parameters and insulin sensitivity has been emphasized as a crucial intervention target in integrated CVD prevention strategies [4, 7].

In the context of cardiovascular pathophysiology, protein S-palmitoylation has been shown to regulate numerous molecular events critically involved in atherosclerosis, myocardial ischemia–reperfusion injury, and heart failure progression. S-palmitoylation refers to the reversible covalent attachment of saturated palmitic acid (C16:0) to cysteine residues via a thioester bond, increasing protein hydrophobicity and membrane association, thereby facilitating enrichment in specialized membrane microdomains such as lipid rafts. This modification influences receptor localization, signal transduction, and protein–protein interactions. Several studies have demonstrated that altered palmitoylation of endothelial proteins affects nitric oxide (NO) production, thus modulating vasodilation and endothelial-protective functions; dysregulation of this process enhances neutrophil adhesion and local oxidative stress, representing an early pathological event in atherogenesis [9, 10]. In cardiomyocytes, palmitoylation of various proteins critically regulates contractile function, electrophysiological properties, and endoplasmic reticulum stress responses; imbalances in these modifications are closely linked to post-ischemic apoptosis and fibrotic processes, thereby driving the development and exacerbation of heart failure [11, 12]. This review aims to systematically elucidate the latest advances in the interplay between protein palmitoylation and lipid metabolism in cardiovascular disease, with a particular focus on their molecular mechanisms, signaling networks, and translational potential.

### Cardiovascular disease and lipid metabolism overview

#### The relationship between lipid metabolic imbalance and cvd/heart diseases

##### Abnormal serum lipid profile and atherosclerotic plaque formation

Atherosclerosis is characterized primarily by the subendothelial deposition of lipids and lipoproteins, the core of which lies in an imbalanced serum lipid profile. Studies have demonstrated that elevated LDL-C, reduced HDL-C, and increased TG are among the principal drivers of atherosclerotic plaque formation [13, 14]. Within the arterial wall, LDL particles are readily oxidized into oxidized LDL (ox-LDL), which induces endothelial cells to express adhesion molecules, promoting monocyte adhesion, transmigration into the intima, differentiation into macrophages, and uptake of ox-LDL to form foam cells, ultimately enlarging the lipid core and evolving into complex lesions [15, 16]. Moreover, small dense LDL (sdLDL) subfractions—more prone to oxidation and endothelial penetration—are particularly abundant at sites of plaque vulnerability and significantly contribute to plaque instability [6]. Meanwhile, serum HDL-C not only mediates reverse cholesterol transport but also exerts antioxidative, anti-inflammatory, and endothelial-protective effects; thus, when HDL-C is low, the capacity to clear cholesterol is compromised, favoring plaque initiation and progression [17, 18]. Multicenter cohort studies have confirmed that serum non–HDL-C levels correlate positively with coronary plaque burden and provide superior predictive value compared to isolated LDL-C measurements; non–HDL-C (total cholesterol minus HDL-C) offers a more comprehensive reflection of atherosclerotic risk [6, 19]. Recent advances in lipidomics have furnished powerful tools for dissecting the lipid composition within atherosclerotic lesions, comparing lipid abundance and species across patients and plaque regions, and elucidating the roles of specific lipid molecules—such as phospholipids, sphingolipids, and oxysterols—in plaque progression [14, 20].

##### NAFLD and cardiovascular risk

NAFLD is defined by excessive triglyceride accumulation in hepatocytes and is closely linked to insulin resistance, chronic low-grade inflammation, and lipid metabolic dysregulation [21, 22]. Epidemiological data indicate that NAFLD prevalence may reach 25% in developed countries and is rising; it is tightly associated with metabolic syndrome, constituting the hepatic manifestation thereof [21, 23]. Meta-analyses show that NAFLD patients face a significantly elevated risk of cardiovascular events—including coronary artery disease (CAD), myocardial infarction, heart failure, and CVD-related mortality—and that this risk correlates positively with NAFLD severity [24, 25]. Crucially, these associations remain significant even after adjusting for traditional risk factors (such as hypertension, diabetes, and obesity), implying that NAFLD itself is an independent cardiovascular risk factor [26, 27]. The underlying mechanisms include: (i) insulin resistance associated with NAFLD elevates serum free fatty acids (FFA), promoting peripheral fatty acid uptake and hepatic TG synthesis, creating lipotoxicity and exacerbating systemic lipid metabolic disturbances, thereby accelerating atherogenesis [28, 29]; (ii) NAFLD provokes a marked increase in proinflammatory cytokines (e.g., TNF-α, IL-6, CRP) and oxidative stress, leading to endothelial dysfunction, smooth muscle proliferation, and a prothrombotic state [28, 30]; (iii) hepatic lipid synthesis abnormalities in NAFLD drive increased VLDL secretion and formation of TG-rich, small dense LDL particles that contribute to atherogenesis [21, 31]. Therefore, early identification and intervention for NAFLD are critical not only for preventing hepatic complications but also as an essential component of both primary and secondary CVD prevention strategies [24, 30].

##### Obesity, metabolic syndrome, and CVD epidemiology

With Westernization of lifestyle and diet, global rates of obesity and metabolic syndrome have risen markedly. According to CDC data, over 40% of U.S. adults are obese [32]; the prevalence of metabolic syndrome is approximately 25% in adults and trending younger in onset [33, 34]. Obesity and metabolic syndrome are independent risk factors for CVD, increasing cardiovascular event risk through mechanisms such as insulin resistance, chronic inflammation, lipid metabolic dysregulation, and endothelial dysfunction [32, 34]. Meta-analyses show that metabolic syndrome patients have nearly twice the risk of cardiovascular events compared with non-metabolic syndrome subjects, alongside significantly higher all-cause and cardiovascular mortality rates [33, 34]; even metabolically healthy obesity is associated with a higher CVD risk than normal-weight individuals [34]. Expansion and dysfunction of adipose tissue in obesity lead to excessive FFA release into the circulation, inducing hepatic and muscular insulin resistance, while also secreting large amounts of proinflammatory cytokines (e.g., TNF-α, IL-6, MCP-1) and reducing anti-inflammatory adipokines (e.g., adiponectin), thereby creating a chronic low-grade systemic inflammatory state that promotes atherosclerosis initiation and progression [34]. Recent large, multicenter, prospective cohort studies have underscored the central role of weight management in preventing cardiovascular events—not only by weight reduction itself but also by reconstructing lipid metabolism, improving insulin sensitivity, and reducing inflammation through diet and exercise interventions, which significantly lower CVD incidence [32]. Furthermore, obesity-associated lipid metabolic disturbances affect serum lipoprotein profiles, such as increasing triglyceride-rich lipoproteins and their remnants, which independently predict CVD risk [35]. In summary, the relationship between obesity, metabolic syndrome, and CVD transcends the additive effects of traditional risk factors, reflecting a systemic metabolic disturbance manifesting at cardiovascular target organs.

#### Etiology and pathogenesis of CVD highlighting the role of lipids

##### Lipids and the multifactorial mechanisms of atherosclerosis formation

Atherosclerosis (AS) develops through multifactorial mechanisms, with lipid metabolism and its derivatives playing a central role. Elevated plasma cholesterol and triglyceride levels—predominantly carried in VLDL, LDL, ox-LDL, chylomicrons, and LDL particles—penetrate the arterial wall and deposit subendothelially, initiating early fatty streak formation in coronary arteries [36]. In plasma, LDL is highly susceptible to oxidative modification by ROS, generating ox-LDL, which accelerates plaque progression via multiple pathways. First, ox-LDL activates endothelial cells (ECs), upregulating adhesion molecules such as ICAM-1 and VCAM-1, thereby recruiting circulating monocytes and lymphocytes to adhere and transmigrate into the intima. Second, ox-LDL is recognized and internalized by macrophage scavenger receptors (e.g., SRA, CD36), causing macrophage transformation into lipid-laden foam cells that accumulate in the subendothelial space to form a soft lipid core [36]. In contrast, HDL exerts inhibitory effects on vascular smooth muscle cells (VSMCs) and macrophages, thereby interrupting or decelerating AS progression. Additionally, high-fat diets or nutrient excess trigger low-grade, chronic systemic inflammation. Studies have shown that in healthy individuals a high-cholesterol diet elevates inflammatory markers such as C-reactive protein (CRP) and serum amyloid A (SAA) [37], while obese patients exhibit systemic inflammation evidenced by elevated circulating CRP and proinflammatory cytokines [38–40]. The mechanism likely involves increased gut permeability after high-fat intake—although lipopolysaccharide involvement is omitted here—and macrophage infiltration into adipose tissue, which secretes TNF-α and IL-6, activating NF-κB signaling in the liver and vasculature and upregulating numerous inflammatory genes, including cytokines and adhesion molecules, thereby promoting atherogenesis [41, 42].

In the liver, elevated circulating TG-rich chylomicron remnants and VLDL remnants are taken up by Kupffer cells via scavenger receptors, which activate hepatic macrophages’ inflammatory response, exacerbating hepatic inflammation and secreting proinflammatory mediators that propagate peripheral vascular inflammation and accelerate AS progression [43]. For example, accumulation of oxysterols, oxidized phospholipids, or oxidized fatty acids within ox-LDL can be recognized by scavenger receptors and activate inflammatory pathways [44].

Within the vasculature, circulating ox-LDL directly induces endothelial activation and morphological changes, is engulfed by infiltrating monocytic cells to form foam cells, amplifies local NF-κB and JNK signaling, and enhances expression of proinflammatory cytokines (e.g., TNF-α, IL-1β, IL-6) and chemokines (e.g., MCP-1), thereby recruiting additional inflammatory cells into the plaque. This further promotes VSMC migration, proliferation, and extracellular matrix synthesis, culminating in fibrous-cap atheroma formation [45–50]. Additionally, hypercholesterolemia can produce cholesterol crystals in the vessel wall, which activate the NLRP3 inflammasome in macrophages, induce IL-1β secretion, exacerbate localized inflammation, and destabilize plaques [51, 52]. TG-enriched lipoprotein remnants also directly interact with circulating monocytes via fatty acid uptake, activating them and promoting their adhesion to the endothelium and subendothelial migration, thus contributing to initial plaque formation [53]. In hypercholesterolemic states, regulatory T cell adhesion and retention in the vessel wall decrease, skewing the effector T cell/regulatory T cell balance toward a proinflammatory phenotype, which further amplifies local inflammation and accelerates atherogenesis; conversely, lowering plasma cholesterol restores T cell subset balance and reduces inflammatory cell content within plaques [54]. Furthermore, fatty acids (FAs) themselves act as inflammatory modulators beyond serving as energy substrates. Saturated fatty acids (e.g., palmitic acid) activate TLRs on macrophages and other cells, inducing NF-κB signaling and exerting proinflammatory effects, whereas long-chain n-3 polyunsaturated fatty acids (e.g., EPA, DHA) bind GPR120 to inhibit NF-κB signaling and exert anti-inflammatory actions [55–63]. Notably, the ratio of n-6 to n-3 FAs often more accurately predicts inflammatory status than the absolute levels of either alone; excess n-6 FAs (e.g., linoleic acid) serve as precursors for proinflammatory eicosanoids, whereas n-3 FAs favor anti-inflammatory lipid mediators, and imbalance predisposes to chronic low-grade inflammation [64, 65].

Peroxisome proliferator–activated receptors (PPARs), liver X receptors (LXRs), and farnesoid X receptor (FXR) form critical bridges between lipid metabolism and inflammation regulation. PPARα, PPARγ, and PPARβ/δ are expressed in endothelial cells, VSMCs, and macrophages and can inhibit proinflammatory gene transcription by direct interaction with NF-κB or by inducing IκB; they also promote macrophage polarization to the anti-inflammatory M2 phenotype, thereby reducing vascular inflammation [66–68]. PPARγ additionally modulates regulatory T cell accumulation in adipose tissue, suppressing obesity-related metabolic inflammation [69]. LXRs are activated by oxysterols and promote cholesterol efflux and reverse transport in macrophages to prevent foam cell formation; they also inhibit LPS-induced proinflammatory gene expression, enhancing inflammatory tolerance [70]. However, synthetic LXR agonists, while attenuating AS in mice, often induce hepatic steatosis and hypertriglyceridemia in humans by upregulating lipogenic transcription factors SREBP-1c and ChREBP [71]. FXR, activated by endogenous bile acids and highly expressed in liver and intestine, regulates bile acid and lipid metabolism and can transrepress LPS-induced inflammation, mitigating hepatic and intestinal inflammation [72, 73]. In AS models, FXR gene knockouts yield conflicting results, but pharmacological FXR activation consistently shows antiatherosclerotic effects, suggesting that FXR agonists may represent novel targets for AS prevention and treatment [74, 75].

Conversely, HDL exerts pronounced antiatherosclerotic and anti-inflammatory functions. HDL primarily facilitates reverse cholesterol transport, shuttling excess cholesterol from peripheral tissues to the liver for biliary excretion, thereby reducing cholesterol burden in the vascular wall [76]. Additionally, HDL inhibits endothelial expression of VCAM-1 and ICAM-1, decreasing monocyte adhesion and infiltration and attenuating local vascular inflammation. Specific lipid components within HDL—especially sphingosine-1-phosphate (S1P)—bind to S1P receptors on endothelial cells and VSMCs to suppress proinflammatory signaling pathways and reduce monocyte–vessel wall interactions, thereby exerting anti-inflammatory effects [77–79]. Under inflammatory conditions, however, HDL functionality can be impaired. In cardiovascular patients, HDL often exhibits dysfunctional properties: both its reverse cholesterol transport capacity and anti-inflammatory characteristics are diminished, thereby reducing its inhibitory effect on plaque development [80].

**Fig. 1:**
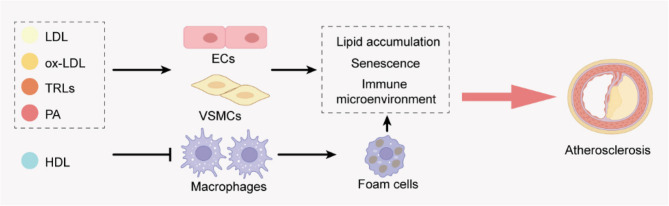
Lipid-Induced Cellular Senescence and Inflammation Drive Plaque Instability in Atherosclerosis. Low-density lipoprotein (LDL), oxidized LDL (ox-LDL), triglyceride-rich lipoproteins (TRLs), and the saturated fatty acid palmitic acid (PA) act on endothelial cells, vascular smooth muscle cells (VSMCs), By contrast, highdensity lipoprotein (HDL) inhibits macrophage foam cell formation. These processes collectively advance atherosclerosis

Figure 1. Lipid-Induced Cellular Senescence and Inflammation Drive Plaque Instability in Atherosclerosis. Low-density lipoprotein (LDL), oxidized LDL (ox-LDL), triglyceride-rich lipoproteins (TRLs), and the saturated fatty acid palmitic acid (PA) act on endothelial cells, vascular smooth muscle cells (VSMCs), and macrophages, inducing cellular senescence and creating an inflammatory microenvironment that fosters plaque instability and advances atherosclerosis (AS).

##### Lipids and myocardial diseases

A healthy heart derives approximately 95% of its ATP from mitochondrial fatty acid β-oxidation (FAO), with only about 5% supplied by anaerobic glycolysis. In metabolic disease states such as obesity and diabetes, circulating nonesterified fatty acids (NEFAs) rise due to insulin resistance or increased adipose lipolysis, driving enhanced FA uptake into cardiomyocytes via transporters such as CD36 [53, 54, 76]. Simultaneously, very-low-density lipoproteins (VLDL) synthesized by the liver and chylomicrons produced by the intestine are hydrolyzed by lipoprotein lipase (LpL) in cardiac capillaries to yield free fatty acids for myocardial oxidation (Fig. 1). LDL and its ox-LDL can also enter cardiomyocytes via CD36 or VLDLR, influencing metabolic regulation [80, 81]. In early heart failure (HF), the myocardium compensates for FAO insufficiency by increasing glucose uptake and utilization. In heart failure with reduced ejection fraction (HFrEF), accelerated anaerobic glycolysis leads to intracellular accumulation of lactate and pyruvate, cytosolic acidification, disruption of contractile protein function, and Ca²⁺ homeostasis, further impairing myocardial contractility [82]. In heart failure with preserved ejection fraction (HFpEF), which often coexists with obesity or type 2 diabetes, mitochondrial dysfunction, insulin resistance, and lipid accumulation lead to metabolic reprogramming and metabolic inflammation [36, 76].

**Fig. 2:**
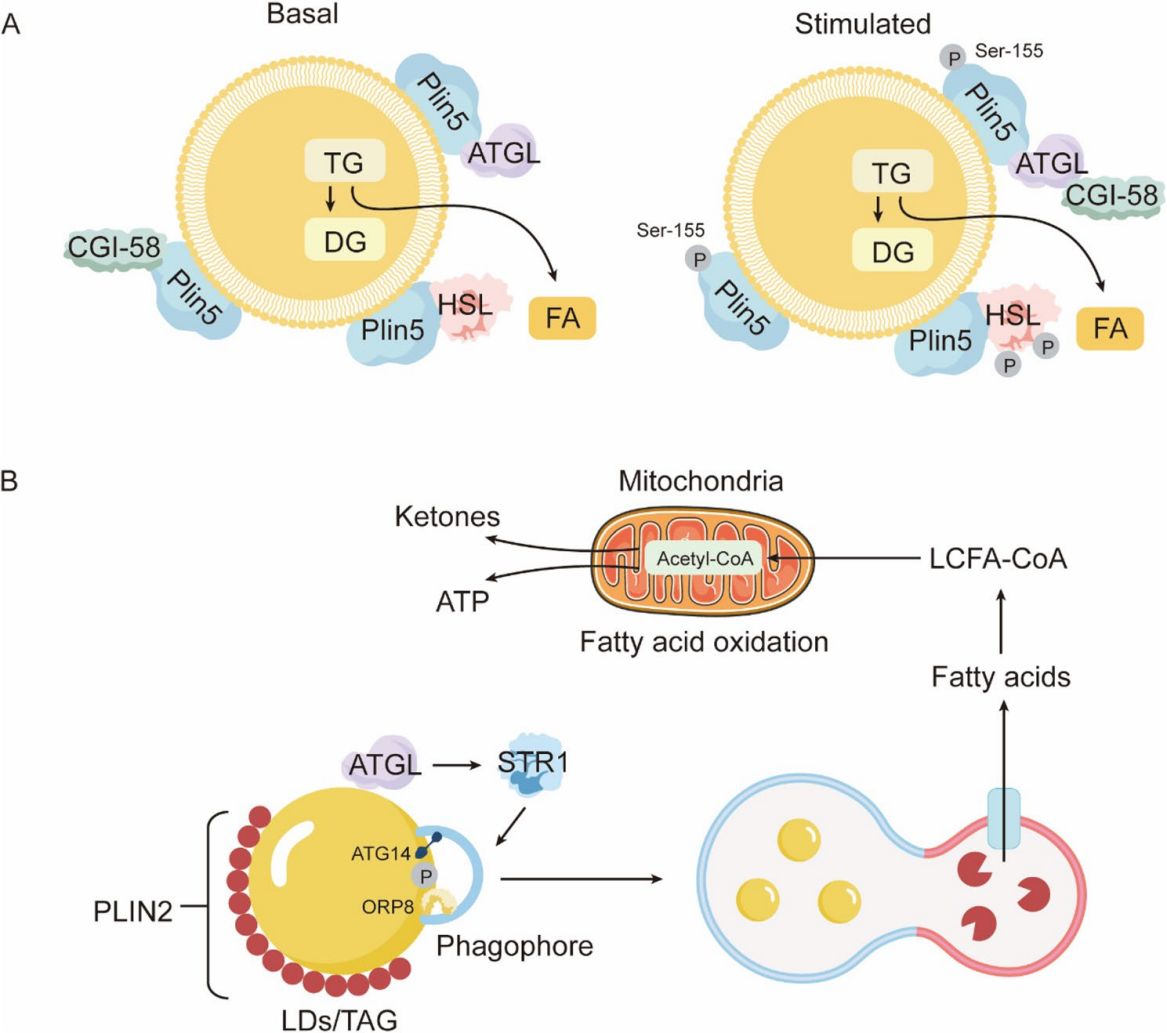
Lipid droplet–associated proteins coordinate lipolysis and lipophagy to fuel mitochondrial fatty acid oxidation. (**A**) Under basal conditions, Plin5 tightly coats lipid droplets (LDs) and sequesters the adipose triglyceride lipase (ATGL) coactivator CGI‑58, restricting ATGL‑ and hormone‑sensitive lipase (HSL)–mediated triacylglycerol (TG)→diacylglycerol (DG) hydrolysis and limiting fatty acid (FA) release. Upon hormonal or β‑adrenergic stimulation, protein kinase A (PKA) phosphorylates Plin5 at Ser‑155, triggering CGI‑58 release and its activation of ATGL, thereby unleashing robust TG and DG hydrolysis and accelerating FA mobilization. (**B**) PLIN2‑coated LDs recruit sirtuin 1–activated ATGL together with autophagy factors ATG14 and oxysterol‑binding protein‑related protein 8 (ORP8) to nucleate a phagophore and initiate lipophagy. Following autolysosome formation, liberated FAs are converted to long‑chain fatty acyl‑CoA (LCFA‑CoA) and transported into mitochondria, where β‑oxidation generates acetyl‑CoA for the tricarboxylic acid (TCA) cycle and ATP production, with excess acetyl‑CoA diverted into ketogenesis

Cardiomyocytes employ CD36-mediated transendothelial uptake of circulating NEFAs or TG derived from chylomicrons/VLDL hydrolyzed by LpL to transport fatty acids into mitochondria for β-oxidation to meet energy demands (Fig. 1). The PPAR family—especially PPARα—is a key upstream regulator of FAO-related genes that enhances expression of CD36 and FAO enzymes in the myocardium to maintain energy balance. However, when PPARα or PPARγ is overactivated, FA uptake by cardiomyocytes can exceed oxidative capacity, leading to a mismatch between lipid uptake and oxidation. Consequently, lipids accumulate intracellularly as TG, diacylglycerols (DAG), ceramides, and cholesterol stored in lipid droplets (LDs), or diffuse into cell membranes and mitochondria, causing lipotoxic injury and ultimately myocardial dysfunction [83–85]. LDs serve not only as lipid reservoirs but also protect against lipotoxicity by sequestering free FAs and cholesterol. LD formation depends on diacylglycerol acyltransferases (DGAT1/2) esterifying DAG and fatty acids to form TG, and is regulated by perilipin family proteins (notably Plin5) that coat LD surfaces. Plin5, by binding adipose triglyceride lipase (ATGL) cofactor CGI-58, inhibits TG lipolysis; upon PKA-mediated phosphorylation of Plin5, CGI-58 dissociates and activates ATGL, initiating TG breakdown to release free FAs for mitochondrial oxidation [86–88] (Fig. 2A). Although myocardial Plin5 overexpression causes substantial LD accumulation, it protects the heart from excessive ROS generation by inhibiting mitochondrial fission (Drp1-dependent) and promoting mitochondrial elongation; conversely, Plin5 deficiency reduces LD content, increases FA entry into oxidative pathways, and exacerbates mitochondrial injury and oxidative stress during ischemia–reperfusion, thereby enlarging infarct size [87]. Meanwhile, Plin2 overexpression in the myocardium markedly promotes LD accumulation; abundant PLIN2 on LD surfaces inhibits AMPK activation, diminishing SIRT1 regulation of autophagy-related proteins (e.g., ATG14 and ORP8). This impairs ATGL recruitment to LDs, reduces phagophore formation and maturation, and decreases autophagy-mediated lipophagy. As a result, stored TG within LDs cannot be timely hydrolyzed to release free FAs for mitochondrial β-oxidation, leading to insufficient LCFA-CoA supply for acetyl-CoA, ATP, and ketone body generation. This impairment weakens myocardial lipid clearance capacity, exacerbates lipotoxicity, and ultimately causes cardiomyocyte dysfunction and structural damage [89] (Fig. 2B). If myocardial LD capacity is insufficient or TG synthesis is impaired (for example, DGAT1 deficiency), unesterified DAG and ceramides accumulate in the cytosol and mitochondria. These intermediates activate signaling pathways such as PKC, MAPK, and JNK, inducing cardiomyocyte apoptosis, inhibiting insulin signaling, causing mitochondrial dysfunction, and elevating ROS, thereby promoting myocardial fibrosis and contractile dysfunction, contributing to the pathophysiology of both HFrEF and HFpEF [90].

Myocardial cholesterol requirements rely predominantly on uptake of circulating LDL and chylomicron remnants, rather than de novo synthesis. Cardiac-anchored LpL not only hydrolyzes TG but also facilitates LDL and cholesterol-rich remnant entry into cardiomyocytes, leading to intracellular cholesterol accumulation and HF development [91, 92]. Myocardial LDL receptor (LDLR) expression is minimal, making CD36 and VLDLR the primary uptake pathways. PCSK9 deficiency leads to increased cardiac CD36 expression, likewise causing cholesterol overload and lipotoxicity in the myocardium [93]. Excess intracellular free cholesterol can be esterified by acyl-CoA: cholesterol acyltransferase (ACAT) in the endoplasmic reticulum or plasma membrane and stored in LDs, or transported to mitochondria and peroxisomes via nonvesicular carriers such as retinol and lipoic acid. However, unesterified free cholesterol can accumulate in mitochondrial or plasma membranes, altering membrane fluidity, increasing rigidity, impeding antioxidant permeation, enhancing proton leak, disrupting inner mitochondrial membrane homeostasis, heightening ROS production, and promoting mitochondrial permeability transition pore (mPTP) opening, which leads to apoptosis, fibrosis, and adverse cardiac remodeling [44, 47].

Cholesterol oxidation products (oxysterols) are highly cytotoxic. For example, 7-ketocholesterol (7-KC) directly damages mitochondrial membranes, causing membrane rupture, cytochrome c release, and autophagy activation; 7-KC also enhances ROS generation via an ATF4-dependent pathway and induces cardiomyocyte apoptosis [94–96]. OxLDL, rich in oxysterols, can upregulate B-type natriuretic peptide (BNP) gene expression in cardiomyocytes, activate the calcium-sensing receptor (CaSR), and promote apoptosis. OxLDL also induces PCSK9 expression in cardiomyocytes, directly impairing contractile function and promoting cell death [97]. The cumulative toxicity disrupts mitochondrial biogenesis (PGC1α-dependent) and dynamics (fusion proteins Mfn1/2 and fission factor Drp1), resulting in reduced fusion, increased fission, further oxidative stress, and accelerated HF progression [98, 99]. Clinical data indicate that when serum cholesterol levels are relatively low in HF patients, prognosis is actually worse, which may be related to malnutrition or cardiac metabolic reprogramming in severe HF, rather than simply the loss of protective substances due to low cholesterol levels [100].

Having shown that lipid dysregulation primes cardiovascular injury, spanning systemic dyslipidaemia as well as local lipotoxicity and inflammation, we now turn to protein S-palmitoylation. This reversible thioacylation of cysteine residues enables cells to convert fluctuations in palmitoylCoA supply into precise changes in protein trafficking, stability and signalling. In this way, palmitoylation links the metabolic disturbances discussed above with the intracellular pathways that govern cardiovascular function.

### Basic concepts and regulatory mechanisms of protein palmitoylation

#### Biochemical process of protein palmitoylation

Cellular lipid metabolic imbalance leads to excessive accumulation of palmitoyl-CoA, supplying abundant acyl donors for protein S‑palmitoylation [101]. Protein palmitoylation is a dynamic and reversible lipid modification of proteins. This modification involves the covalent attachment of medium- or long-chain fatty acids such as palmitic acid to the thiol group of specific cysteine residues in target proteins. Although palmitate (C16:0) is the most common acyl donor, other acyl chains such as stearate (C18) can also serve as donors; thus, this modification is sometimes broadly referred to as S-acylation [102, 103]. The post-translational addition of palmitate is primarily catalyzed by a family of enzymes known as palmitoyltransferases (PATs). In mammals, these enzymes are membrane proteins that share a highly conserved DHHC (Asp-His-His-Cys) catalytic motif; they are collectively referred to as ZDHHC proteins. ZDHHC family members localize to membranes of various organelles, including the endoplasmic reticulum, Golgi apparatus, and plasma membrane [104]. Structurally, each ZDHHC enzyme contains four transmembrane domains, with the conserved DHHC catalytic site located in the cytosolic loop between the second and third transmembrane helices. Differences among family members arise primarily in their N- and C-terminal sequences and in the presence of additional functional domains such as ankyrin repeats (e.g., ZDHHC13 and ZDHHC17), PDZ domains (e.g., ZDHHC5 and ZDHHC8), or SH3 domains (e.g., ZDHHC6) [105–107].

One crucial feature of palmitoylation is its reversibility, which is mediated by palmitoyl protein thioesterases (APTs). The APT family, including APT1, APT2, and α/β-hydrolase domain-containing proteins (ABHD family), hydrolyzes the thioester bond, releasing free fatty acid and restoring the unmodified cysteine residue on the protein. The balance between palmitoylation and depalmitoylation confers high dynamism to this modification, enabling tight regulation of protein function, subcellular localization, and involvement in cellular signaling and metabolism [108–110].

#### Substrate recognition and reaction steps of palmitoylation

Palmitoylation of a given protein substrate depends on specific recognition by a ZDHHC enzyme. Substrate recognition does not rely solely on the presence of a cysteine residue; rather, it is determined by multiple factors, including the primary amino acid sequence surrounding the target cysteine, membrane-targeting signals, and the three-dimensional conformation of the substrate. Some ZDHHC enzymes, such as ZDHHC17, exhibit high selectivity for particular protein substrates, whereas others, such as ZDHHC3 and ZDHHC7, display broader substrate spectra [111, 112]. Sites of palmitoylation are often located near membrane-binding regions or in proximity to transmembrane domains. These target cysteines frequently feature conserved local motifs such as adjacent basic or hydrophobic residues that facilitate precise recognition and efficient catalysis by ZDHHC enzymes [113–115].

**Fig. 3:**
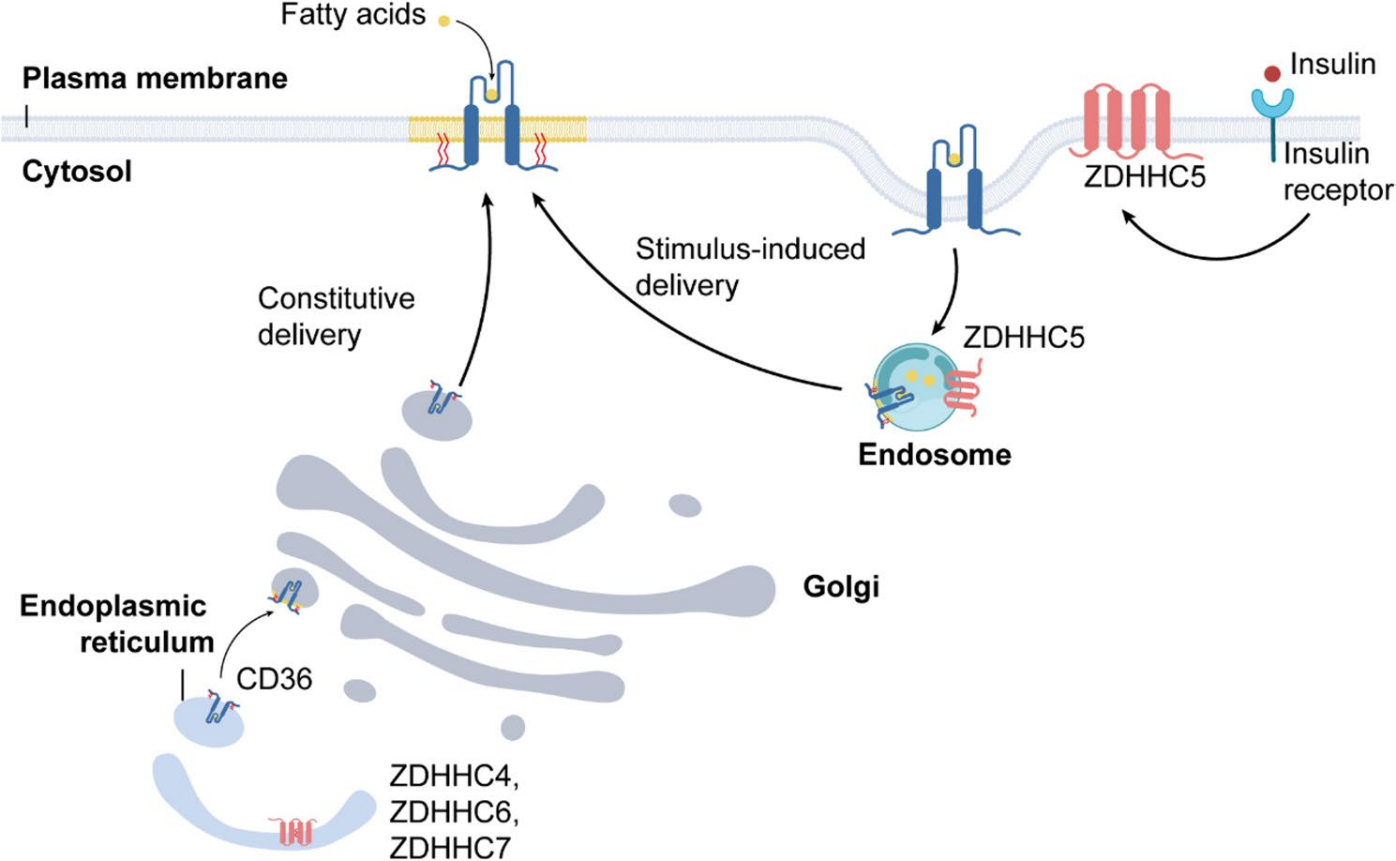
Dynamic palmitoylation cycle and trafficking of the fatty-acid transporter CD36. Newly synthesized CD36 is first S-acylated by ZDHHC4/6/7 in the endoplasmic reticulum and packaged into COPII vesicles for constitutive delivery through the Golgi to the plasma membrane. At the surface, ZDHHC5 undergoes autoacylation with palmitoyl-CoA and then transfers the acyl group to CD36, driving its partitioning into cholesterol- and sphingolipid-rich membrane nanodomains where high-affinity fatty-acid binding occurs. Upon fatty-acid engagement, APT1 rapidly depalmitoylates CD36, triggering endocytosis into early endosomes and subsequent delivery back to the Golgi or ER for recycling. Insulin receptor activation further recruits ZDHHC5 to promote stimulus-induced CD36 palmitoylation and surface retention. This two-step catalytic mechanism—autoacylation of ZDHHC5 followed by trans-thioesterification to CD36—is dynamically balanced by APT1 to allow rapid modulation of fatty-acid uptake in response to metabolic and hormonal cues

Figure 3 shows the catalytic mechanism of palmitoylation proceeds in two steps (Fig. 3). First, the catalytic cysteine of the ZDHHC enzyme undergoes autoacylation by reacting with an acyl-CoA donor such as palmitoyl-CoA, forming an enzyme–palmitate thioester intermediate. Second, the acyl group is transferred from the enzyme to the thiol side chain of the substrate cysteine via a trans-thioesterification reaction. Intracellularly, this cycle of palmitoylation and depalmitoylation is tightly regulated and rapid, allowing palmitoylation levels to respond quickly to changes in extracellular or intracellular cues such as activation of signaling pathways or cellular stress [116, 117]. The kinetic equilibrium between palmitoylation and depalmitoylation is critical for dynamic control of signaling cascades. For instance, growth factors like insulin can rapidly activate specific ZDHHC enzymes such as ZDHHC5, elevating palmitoylation of downstream substrates and swiftly modulating signaling outputs [118, 119]. A representative example is the fatty acid transporter CD36. Initial palmitoylation of CD36 in the endoplasmic reticulum is catalyzed by multiple ZDHHC enzymes including ZDHHC4, ZDHHC6, and ZDHHC7. Palmitoylated CD36 then traffics via the Golgi to the plasma membrane, where ZDHHC5 further palmitoylates it, promoting its partitioning into lipid nanodomains—specialized microdomains enriched in cholesterol and sphingolipids—and facilitating high-affinity fatty acid binding and uptake [120, 121]. Upon fatty acid binding, plasma membrane–localized APT1 rapidly depalmitoylates CD36, triggering endocytosis and internal delivery of fatty acids [122, 123]. Concurrently, insulin signaling–activated ZDHHC5 stimulates translocation of an intracellular CD36 pool to the plasma membrane, sustaining efficient fatty acid uptake [118–120]. Thus, the dynamic cycle of palmitoylation and depalmitoylation precisely regulates CD36 function, localization, and downstream lipid uptake pathways.

#### Roles of palmitoylation in subcellular targeting and signal transduction

One of the most prominent biological effects of palmitoylation is to increase protein affinity for cellular membranes or specific subcellular membrane domains, known as membrane anchoring. Because of the hydrophobic nature of the added fatty acid chain, palmitoylation markedly enhances a protein’s interaction with lipid bilayers, especially cholesterol- and sphingolipid-rich microdomains known as lipid rafts. This selective membrane targeting is essential for proper assembly of signaling platforms and for the function of cell-surface receptors [124]. Beyond membrane localization, palmitoylation also regulates protein–protein interactions, modulating the assembly and disassembly of signalosome complexes. For example, calcium-signaling proteins such as STIM1 and ORAI1 require palmitoylation for assembly and function at ER–plasma membrane junctions, ensuring precise control of store-operated calcium entry [125, 126].

Within intracellular signaling networks, palmitoylation exerts pivotal regulatory effects. Numerous studies have shown that palmitoylation influences key cascades such as the phosphoinositide 3-kinase/AKT pathway, mitogen-activated protein kinase signaling, and AMP-activated protein kinase activity and duration. For instance, the transcription factor STAT3 undergoes palmitoylation by ZDHHC7 to achieve membrane association; upon stimulation by growth factors or interferons, depalmitoylation by APT2 liberates STAT3, allowing its nuclear translocation and transcriptional activation of target genes [127].

### Interplay between lipid metabolism and protein palmitoylation

**Fig. 4:**
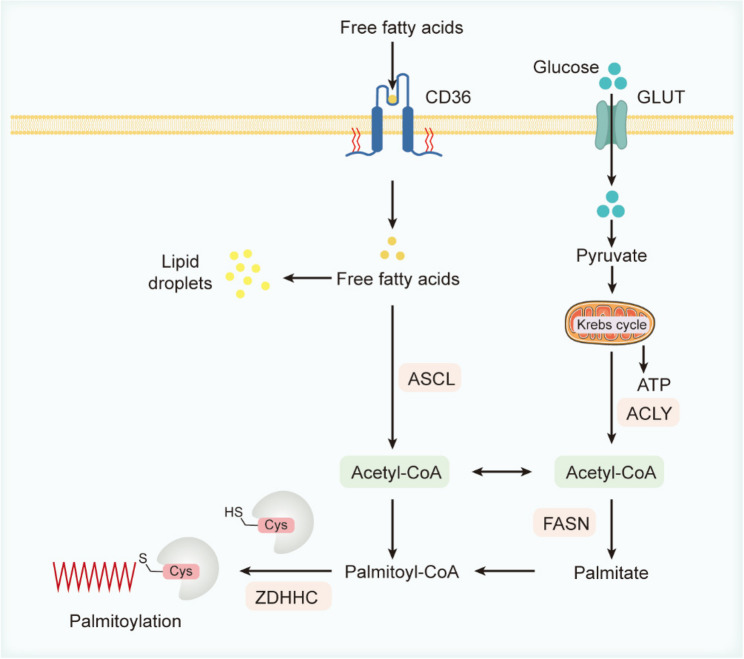
Metabolic pathways supplying palmitoyl-CoA for protein palmitoylation. FFAs taken up via CD36 are activated by ACSLs to form acyl-CoAs. Glucose enters via GLUT4, is converted to citrate in the TCA cycle, and cleaved by ACLY to acetyl-CoA, which ACC carboxylates to malonyl-CoA. FASN then uses acetyl- and malonyl-CoA to synthesize palmitate, which ACSLs convert to palmitoyl-CoA. Palmitoyl-CoA serves as the direct acyl donor for ZDHHC-mediated thioesterification of protein cysteines

Protein S‑palmitoylation is inherently dependent on intracellular palmitoyl‑CoA levels, which are determined by the coordinated activities of fatty acid synthase, long‑chain acyl‑CoA synthetases and the CD36 fatty acid transporte [128]. As shown in Fig. 4, lipid metabolism and protein S-palmitoylation are intricately connected (Fig. 4). As a critical post-translational modification, S-palmitoylation regulates intracellular signal transduction, protein targeting, and functional control. In addition to the well-characterized protein acyltransferases (PATs) and acyl protein thioesterases (APTs), multiple key enzymes and metabolic intermediates within the fatty acid metabolic network significantly influence the dynamics of palmitoylation. Recent studies have progressively revealed that fatty acid synthase (FASN) and its downstream products—palmitate (PA) and palmitoyl-CoA—play pivotal roles in regulating protein palmitoylation in vivo [129]. Under physiological conditions, intracellular concentrations of fatty acids and their derivatives are tightly regulated, with palmitoyl-CoA levels varying between approximately 100 nM and 10 µM across different tissues or metabolic states [130]. Notably, palmitoyl-CoA concentration not only determines donor availability for the palmitoylation reaction but also may act as a feedback signal that influences lipid synthesis pathways—for example, modulating the catalytic activity of FASN. This metabolic feedback regulation underscores the interdependence between cellular lipid metabolism and palmitoylation.

At the molecular level, lipid metabolism impacts protein palmitoylation primarily by controlling the generation and supply of acyl donors. Cytosolic acetyl-CoA is generated from citrate via ATP-citrate lyase (ACLY), and then acetyl-CoA is carboxylated by acetyl-CoA carboxylase (ACC) to form malonyl-CoA. Both substrates feed into FASN, which synthesizes PA [129]. Palmitate is subsequently converted to palmitoyl-CoA by long-chain acyl-CoA synthetases (ACSLs), and palmitoyl-CoA serves as the direct acyl donor for protein palmitoylation. FASN activity thus constitutes a critical regulatory node in controlling the overall level of protein palmitoylation. For example, FASN overexpression markedly increases intracellular palmitate production, thereby elevating palmitoylation of target proteins, whereas pharmacological or genetic inhibition of FASN reduces palmitoylation of specific substrates—such as EGFR, PD-L1, and MYD88—ultimately affecting their stability, subcellular localization, and physiological functions [131, 132].

Beyond endogenous fatty acid synthesis, exogenous fatty acid uptake and utilization also play central roles in palmitoylation regulation. CD36, a transmembrane fatty acid transporter, mediates uptake and endocytosis of circulating free fatty acids and is essential for lipid homeostasis and energy metabolism in multiple tissues [133]. In mice, CD36-dependent fatty acid uptake accounts for roughly 50% of total fatty acid uptake in adipose tissue and skeletal muscle; CD36-deficient mice exhibit significantly reduced fatty acid uptake and concomitant increases in plasma free fatty acids, cholesterol, and triglycerides [134]. Under conditions of limited fatty acid availability or CD36 inhibition, palmitoylation of specific proteins—such as MYD88—is markedly reduced, indicating that CD36-mediated fatty acid transport and metabolism directly modulate protein palmitoylation reactions [129]. Key lipid metabolic enzymes, including ACSLs and short-chain acyl-CoA synthetases (ACSSs), similarly influence palmitoylation by regulating the intracellular pool of acyl-CoA. The ACSL family catalyzes the activation of long-chain fatty acids to form acyl-CoA. Under various physiological and pathological conditions—such as cancer or inflammatory states—ACSL expression increases, boosting palmitoyl-CoA supply and promoting higher levels of protein palmitoylation. For instance, palmitoylation of Wnt2B, endothelial nitric oxide synthase (eNOS), and the G-protein subunit Gsα has been shown to correlate positively with ACSL expression; treatment with the ACSL inhibitor triacsin C effectively reduces palmitoylation of these proteins [135, 136]. Moreover, members of the ACSS subfamily—such as ACSS2—are highly upregulated in cisplatin-resistant bladder cancer cells; inhibition of ACSS2 activity reduces de novo fatty acid synthesis by over 60%, and concurrent decreases in palmitoylation levels impair cell migration and proliferation processes regulated by specific palmitoylated proteins [131].

The chain length and saturation of fatty acid substrates, as well as their derivatives, further modulate palmitoylation specificity. For example, palmitic acid can be interconverted with stearic acid; intracellular stearoyl-CoA can also serve as a substrate for protein lipidation. The transcription factor RFX3 preferentially undergoes lipidation by C18 fatty acids, and the degree of its acylation directly influences its dimerization and transcriptional activity [137]. Importantly, these lipid-derived metabolites can directly contribute to auto-palmitoylation of ZDHHC family enzymes themselves, thereby generating palmitoylation cascades that further regulate protein stability and enzymatic activity [105].

### Protein s‑palmitoylation as a master switch in cardiovascular disease

Building on the established crosstalk between lipid metabolic pathways and palmitoylation machinery, this section examines how cycles of palmitoylation and depalmitoylation function as a regulatory switch in cardiovascular pathology. Reversible S‑palmitoylation has emerged as a pivotal post‑translational modification that choreographs the progression of cardiovascular disease [138]. By dictating the membrane targeting and proteostability of critical effectors, palmitoylation governs lipid trafficking, nitric‑oxide bioavailability, programmed cell death pathways and ion‑channel electrophysiology, thereby exerting decisive control over atherosclerosis, arrhythmogenesis, myocardial remodeling and vascular tone or dilation [139]. Table 1 synthesizes the principal palmitoylated proteins, their mechanistic roles and potential therapeutic entry points across cardiovascular disorders.

**Table 1:** S-palmitoylation enzymes/depalmitoylases of cardiovascular-related proteins and their functional impact

Cardiovascular disease	Protein name	Palmitoylating/depalmitoylating enzymes	Pathological effect	References
Coronary artery disease	CD36	zDHHC4, zDHHC5	Palmitoylation by zDHHC4/5 increases CD36’s hydrophobicity and anchors it in plasma-membrane microdomains for efficient free fatty-acid capture.	[120]
		APT1	APT1 depalmitoylates CD36, recruits SYK, phosphorylates JNK/VAV, and triggers receptor-mediated endocytosis of the CD36–fatty-acid complex for intracellular transport and utilization.	[123]
		zDHHC4	TGR5 loss drives zDHHC4-mediated CD36 palmitoylation, boosting its membrane localization and cardiac fatty acid uptake, and causing lipid accumulation.	[174]
	eNOS	DHHC-21	S-palmitoylation by zDHHC21 promotes eNOS Golgi–caveolae targeting, enabling Ca²⁺-triggered NO production and vascular function.	[151]
	PLCβ1	DHHC-21	DHHC21 palmitoylates PLCβ1, promoting its membrane targeting and activation to amplify inflammatory endothelial leakiness and leukocyte adhesion.	[152]
	H-Ras	APT1	APT1 depalmitoylates H-Ras, triggers RAF–MEK–ERK signaling, upregulates ICAM-1/VCAM-1 and cytokines, and accelerates plaque formation.	[187]
	ADP ribosylation factor like GTPase 13B	-	ARL13B S-palmitoylation restores endothelial cilia and reduces atherosclerosis progression.	[10]
	STING	-	S-palmitoylation of STING promotes its interaction with STXBP2, SNARE complex assembly, and granule secretion, thereby leading to thrombosis.	[155]
	GSDMD	ZDHHC14	ZDHHC14-mediated S-palmitoylation of GSDMD at Cys192 enhances N-terminal membrane targeting and pore formation, exacerbating cardiomyocyte pyroptosis and AMI progression.	[156]
Cardiac Arrhythmias	NCX1	ZDHHC5, APT1	NCX1 S-palmitoylation drives dimer rearrangement and lipid-domain targeting to enhance XIP binding, inhibit exchange activity, and lower intracellular Ca²⁺; APT1 depalmitoylates NCX1 to restore its conformation and function.	[164]
	Nav1.5	-	Palmitoylation at the Cys981 site of Nav1.5 enhances its sensitivity, resulting in increased cardiomyocyte excitability.	[163]
	Kv1.5	-	Palmitoylation of the Kv1.5 potassium channel at Cys593 enhances membrane potassium currents, increasing the risk of arrhythmia.	[166]
	Cav1.2	-	Palmitoylation of the α1C subunit enhances Cav1.2 membrane anchoring, boosts Ca²⁺ influx, triggers SR calcium release, and drives myofilament contraction.	[167, 168]
	JPH1–JPH4	DHHC	S-palmitoylation of JPH2 enriches it in plasma membrane lipid rafts and, via raft association, protects it from depalmitoylation, thereby doubly stabilizing ER/SR–PM junctions.	[169]
Cardiomyopathies	Gαs, Gαi	DHHC5	Gαs/Gαi palmitoylation anchors them in caveolar rafts, driving downstream signaling for cardiac excitation–contraction coupling.	[119]
	Rac1	zDHHC3/zDHHC7	Golgi zDHHC3/7–mediated Rac1 palmitoylation enhances its signaling and drives adverse cardiac remodeling.	[138]
	Rab3gap1	zDHHC9	Rab3gap1 palmitoylation dissociates it from Rab3a, raising Rab3a-GTP and Rab3a⁺ vesicles, and impairing ANP exocytosis.	[170]
	NLRP3	zDHHC12	Palmitoylation of NLRP3 promotes inactivation of the NLRP3 inflammasome, thereby alleviating septic myocardial injury.	[175]
Vascular Dysfunction	R-Ras	APT1	APT-1 loss blocks R-Ras palmitoylation, trapping it at the plasma membrane and impairing trafficking, fibronectin processing, junction integrity, lumen formation, and recovery.	[177]
	α1D adrenoceptor	ZDHHC21	ZDHHC21 palmitoylates and complexes with the α1D-adrenergic receptor; ZDHHC21 deficiency lowers vascular tone, causing hypotension and tachycardia in vivo.	[178]
	Gpx1	PPT1	PPT1 decreases GPx1 protein stability via depalmitoylation, thereby inhibiting angiogenesis.	[179]
	Gαq	zDHHC3, zDHHC7	zDHHC3 and zDHHC7 mediate Gαq palmitoylation, altering its association with the thromboxane receptor and influencing the progression of hypoxic pulmonary hypertension.	[180]

#### Coronary artery disease — from lipid deposition to thrombus formation

Coronary artery disease (CAD) ultimately underpins myocardial infarction, heart failure, ischemic cardiomyopathy and stroke [140–142]. Its pathological substrate is atherosclerosis [143], driven by dysregulated lipid–cholesterol homeostasis and endothelial dysfunction. Macrophages, vascular endothelial cells and vascular smooth‑muscle cells orchestrate plaque initiation and progression [144, 145]. Accumulating evidence indicates that S‑palmitoylation integrates the entire “lipid deposition-endothelial injury-thrombosis” cascade by fine‑tuning CD36‑dependent lipid uptake, eNOS‑mediated nitric‑oxide synthesis and GSDMD‑initiated pyroptosis [128, 146, 147]. Elucidating how palmitoylation modulates individual steps of CAD pathogenesis could catalyze precision‑targeted interventions.

**Fig. 5:**
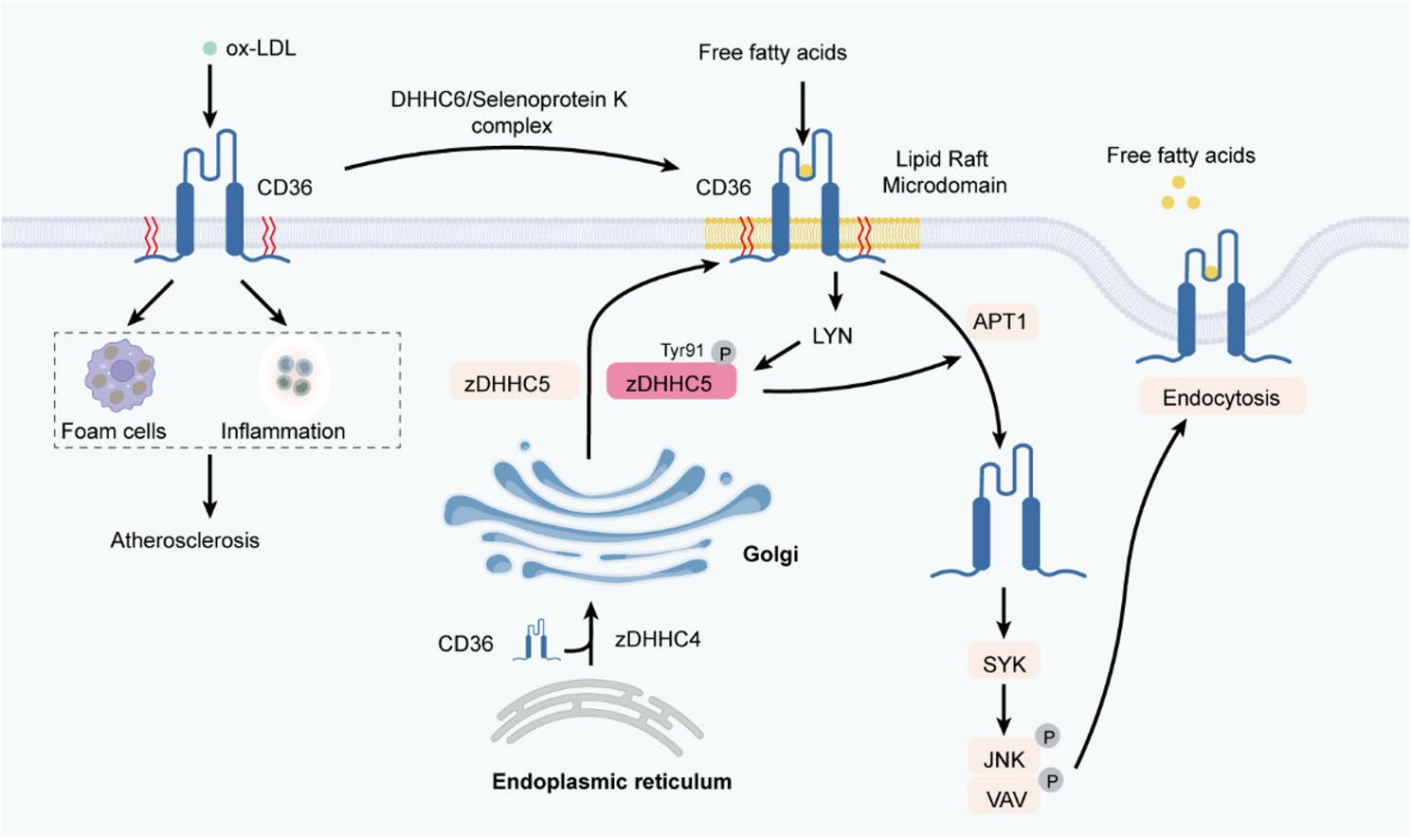
Palmitoylation-Dependent Regulation of CD36 in Oxidized Lipoprotein Uptake and Atherosclerosis Progression. CD36 recognizes and internalizes oxidized low-density lipoprotein (ox-LDL), triggering inflammation and promoting foam-cell formation, thereby accelerating atherosclerosis. Oxidized high-density lipoprotein (ox-HDL) can further enhance palmitoylation of cytoplasmic cysteine residues on CD36 by activating the DHHC6/selenoprotein K complex, causing rapid enrichment of CD36 in cholesterol- and sphingolipid-rich lipid-raft microdomains. CD36 undergoes an initial palmitoylation in the endoplasmic reticulum mediated by zDHHC4, followed by terminal palmitoylation in the Golgi apparatus mediated by zDHHC5, which increases its hydrophobicity, secures its anchoring in plasma-membrane microdomains, and facilitates capture of free fatty acids. Once fatty acids bind to palmitoylated CD36 on the plasma membrane, the Src-family kinase LYN is activated and phosphorylates Tyr91 of zDHHC5, suppressing its palmitoyl-transferase activity and promoting removal of the palmitoyl group from CD36 by APT1. Depalmitoylated CD36 then recruits the tyrosine kinase SYK, which subsequently phosphorylates downstream JNK and VAV proteins, initiating a receptor-mediated endocytic process

At the macrophage plasma membrane, CD36 senses and internalizes oxidized LDL (oxLDL), launching inflammatory signaling and foamcell generation that accelerate atherosclerosis (Fig. 5) [148, 149]. CD36 activity is orchestrated by a palmitoylation–depalmitoylation cycle mediated by zDHHC4/zDHHC5 and the thioesterase APT1. Initial palmitoylation by zDHHC4 in the endoplasmic reticulum and terminal palmitoylation by zDHHC5 in the Golgi markedly enhance hydrophobicity, tethering CD36 to cholesterolrich microdomains and enabling highaffinity fattyacid capture [120]. Upon fattyacid engagement, the Srcfamily kinase LYN phosphorylates Tyr91 of zDHHC5, attenuating its transferase activity and permitting APT1driven depalmitoylation. Depalmitoylated CD36 then recruits the tyrosine kinase SYK, which phosphorylates JNK and VAV to instigate receptormediated endocytosis and downstream trafficking of the CD36–lipid complex [123]. Under lipid overload—triggered by a high fat diet or exposure to excess free fatty acids—CD36 dependent fatty acid uptake relies on the receptor’s dynamic palmitoylation and depalmitoylation cycle. Pharmacological inhibition of LYN/SYK mediated endocytosis or the DHHC5/APT1 controlled palmitoylation switch markedly limits lipid droplet expansion [123]. Likewise, deletion of DHHC4 or DHHC5 reduces CD36 palmitoylation and impairs fatty acid uptake [120]. Oxidized HDL (oxHDL) further augments CD36 palmitoylation via activation of the DHHC6–SelenoproteinK complex, concentrating CD36 within lipid rafts alongside caveolin-1 and Lyn/Fyn. Lyn/Fyntriggered phosphorylation of JNK and VAV remodels actin and accelerates endocytosis, thereby promoting efficient fattyacid uptake [150]. Pharmacological inhibition of CD36 palmitoylation with 2bromopalmitate sharply reduces CD36 surface expression and macrophage oxHDL uptake [150].

Endothelial nitricoxide synthase (eNOS) undergoes palmitoylation by zDHHC21, which promotes its localization to the Golgi and caveolae and ensures rapid NO production upon Ca²⁺ stimulation [151]. Loss of zDHHC21 diminishes eNOS palmitoylation, membrane targeting and NO release. In zDHHC21null mice, endothelial barrier integrity is compromised; zDHHC21dependent palmitoylation of PLCβ1 exacerbates inflammatory permeability and leukocyte adhesion, whereas pharmacological inhibition of zDHHC21 or blockade of PLCβ1 palmitoylation preserves endothelial function [152]. Within atherosclerotic milieus, miR-138 downregulation elevates APT1 expression; APT1 depalmitoylates HRas, relocates it to the plasma membrane and activates the RAF–MEK–ERK cascade, inducing ICAM-1/VCAM-1 and proinflammatory cytokines in endothelial cells. Endothelial exosomes (BiNPs) conveying APT1 mRNA deliver this signal to macrophages, driving M1 polarization and magnifying inflammation to accelerate plaque development [153]. Loss of palmitoylation on the ciliary GTPase ARL13B precipitates cilia shedding and worsens atherosclerosis [10]. Triglyceride accumulation alters freefattyacid composition in endothelial membranes, suppresses ARL13B palmitoylation and jeopardizes ciliary integrity, whereas dietary palmitate replenishment restores cilia and retards disease progression [10].

Palmitoylation of thrombosisassociated proteins is indispensable for platelet activation [154]. Circulating cGAMP generated during sepsis binds platelet STING, whose palmitoylation facilitates STXBP2 engagement, SNARE assembly and granule exocytosis, culminating in septic thrombosis [155]. In myocardial infarction tissue and hypoxic cardiomyocytes, global palmitoylation increases in parallel with pyroptosis [156]. zDHHC14 catalyzes Spalmitoylation of GSDMD at Cys192, fostering pore formation and accelerating pyroptotic cardiomyocyte death during acute myocardial infarction [156]. A palmitoylationdeficient estrogenreceptorα variant (ERαC451A) heightens the cardioprotective response to estrogen, retarding atherosclerosis and angiotensinIIinduced hypertension, underscoring the role of ERα palmitoylation in vascular remodeling [157]. In apolipoprotein E knockout mice (ApoE/) fed a Westerntype highfat diet, lipid overload markedly activates the STING pathway in arterialwall macrophages. Administration of the STINGspecific smallmolecule antagonist C-176 significantly decreases atherosclerotic plaque burden and lowers thrombotic risk [158]. By covalently binding to Cys88 and Cys91 of STING, C-176 blocks stimulusinduced Spalmitoylation, thereby suppressing downstream TBK1/IRF3 signaling and the expression of proinflammatory factors [159]. Moreover, a shortterm diet rich in palmitic acid strongly elevates Spalmitoylation of GSDMD at Cys192 in the myocardium, exacerbating pyroptosis and infarct size after myocardial infarction; the depalmitoylation antagonist disulfiram reverses these injuries and improves cardiac function by preventing palmitoylation at this site [156].

#### Cardiac arrhythmias — fine‑tuning the electrical apparatus

Arrhythmogenesis reflects aberrant ionchannel conductance, extracellularmatrix remodeling, inflammation and disruption of excitation–contraction coupling [160, 161]. Palmitoylation modulates Na⁺, K⁺ and Ca²⁺ channels, thereby shaping arrhythmic susceptibility [162]. In lipid overload models, saturated fatty acids directly drive aberrant S-palmitoylation of cardiac ion channels and provoke electrophysiologic imbalance. In adult rat cardiomyocytes incubated with 10 µM palmitic acid for 24 h, Nav1.5 shows a marked increase in S-palmitoylation, leading to significant prolongation of action potential duration and the appearance of early afterdepolarizations, indicating that excess fatty acids may raise arrhythmia susceptibility by augmenting the late sodium current [163]. In neonatal rat ventricular myocytes, exposure to 20 µM palmitic acid for only 4 h markedly elevates membrane S-palmitoylation of NCX1, strengthens XIP dependent inactivation, and increases cytosolic Ca²⁺ load [164]. Multiple Na⁺/K⁺ATPase subunits (α, β, FXYD) are palmitoylated. In cardiomyocytes the α3 subunit, phospholemman, suppresses pump activity when palmitoylated, perturbing intracellular Na⁺ and indirectly Ca²⁺ via NCX1 to modulate contractility [165]. zDHHC5 palmitoylates NCX1 at Cys739, inducing dimeric rearrangement, lipidraft affinity and XIP binding that dampen exchanger activity and lower cytosolic Ca²⁺; APT1mediated depalmitoylation restores exchanger function [164]. Palmitoylation at Cys981 augments Na_v_1.5 sensitivity, enhancing excitability [163]. For the atrial Kv1.5 K⁺ channel, Cys593 palmitoylation amplifies surface current and elevates arrhythmic risk [166]. Likewise, Ltype Ca²⁺ channel Cav1.2 α1C palmitoylation stabilizes membrane anchoring and Ca²⁺ influx, triggering RyRmediated Ca²⁺ release and contraction [167, 168]. Junctophilins (JPH1–4) undergo dual palmitoylation within the MORN domain and Cterminal TMD, fortifying ER/SR–plasmamembrane junctions essential for excitation–contraction coupling [169].

#### Cardiomyopathies — remodeling, signaling and metabolic regulation

Through regulation of apoptosis, inflammation, structural remodeling and energy metabolism, palmitoylation is inextricably linked to diverse cardiomyopathies. zDHHC5mediated palmitoylation of Gαs and Gαi anchors them to caveolae, facilitating βadrenergic signaling; dysregulation exacerbates βAR desensitization and contractile dysfunction in heart failure [119]. zDHHC3/7 drive hypertrophy via Rac1 palmitoylation at the Golgi, propelling maladaptive remodeling [138]. Under pressure overload, natriuretic peptides relieve hemodynamic stress; however, zDHHC9dependent palmitoylation of Rab3gap1 disrupts Rab3a compartmentalization, elevates Rab3aGTP and impairs atrialnatriureticpeptide exocytosis, thereby contributing to dilatedcardiomyopathyrelated failure [170].

In diabetic cardiomyopathy palmitoylation modulates insulin signaling [171], fattyacid metabolism [172] and membraneprotein function. Palmitoylation of SNAP23, GLUT4 and CD36 governs lipidinduced insulin resistance [173]. Deletion of the bileacid receptor TGR5 upregulates zDHHC4mediated CD36 palmitoylation, intensifying fattyacid uptake and precipitating cardiac lipid overload [174]. Vaccarin enhances zDHHC12driven NLRP3 palmitoylation, inactivating the inflammasome and ameliorating septic myocardial injury [175].

#### Vascular dysfunction — from vascular tone to angiogenesis and pulmonary hypertension

In a saturated fatty acid overload model, ApoE^⁻/⁻^ mice received intraperitoneal injections of palmitic acid at 10 mg/kg for ten weeks, resulting in higher blood pressure, weaker acetylcholine mediated vasodilation, and larger aortic plaques. Mechanistic studies showed that palmitic acid triggers zDHHC13 dependent S-palmitoylation of PKM2 at Cys31, which suppresses endothelial glycolysis and barrier function. Endothelial specific expression of the depalmitoylation resistant mutant PKM2 C31S completely reversed these vascular injuries [176]. Pharmacological inhibition of palmitoylation with 2 bromopalmitate alleviated, whereas enhancement with ML349 exacerbated these lesions [176]. Endothelial APT1 is indispensable for RRas depalmitoylation and vessel maturation; APT1 deficiency or hyperglycemia immobilizes RRas at the plasma membrane, derailing trafficking, fibronectin processing, junctional adhesion, lumen formation and postischemic recovery. Introducing a hydrophilic linker into RRas restores mobility and vascular integrity, highlighting metabolic regulation of endothelial palmitoylation [177]. zDHHC21 palmitoylates the α1Dadrenergic receptor and forms a functional complex; zDHHC21 insufficiency attenuates receptor activity, reduces systemic vascular resistance and induces hypotension with reflex tachycardia [178]. Dynamic palmitoylation of Gpx1 at Cys76/113 orchestrates angiogenesis, as PPT1mediated depalmitoylation destabilizes Gpx1 and suppresses neovessel formation [179]. In persistent pulmonary hypertension of the newborn (PPHN), hypoxia intensifies zDHHC3/7dependent palmitoylation of Gαq at Cys9/10, anchoring it to the plasma membrane, coupling it to TPα receptors and amplifying PLCmediated Ca²⁺ mobilization and vasoconstriction. Inhibiting palmitoylation selectively blunts hypoxic, but not normoxic, pulmonaryartery contraction, pinpointing Gαq palmitoylation as a viable target in PPHN [180].

### Clinical translation of S-palmitoylation — biomarkers, targets and therapeutic modalities

Recent investigations position Spalmitoylation as a dualpurpose clinical tool: a prognostic biomarker and an actionable therapeutic target across multiple pathologies [181]. Protein acyltransferases (PATs), acylprotein thioesterases (APTs) and palmitoylprotein thioesterases/esterases (PPTs) constitute the enzymatic triad that orchestrates this reversible lipidation; pharmacologically modulating their activity therefore offers a plausible route to correct palmitoylation imbalancedriven disorders [182]. Within cardiomyocytes, palmitoylation operates as a reversible switch shaping electrophysiology, metabolism and signalling. Structural and functional studies over the past decade tie disruption of the palmitoylation network to lethal arrhythmias, heart failure and metabolic cardiomyopathy, thereby enlarging the druggable landscape for precision cardiovascular intervention. The latest advances in palmitoylationbased regulation of heart and vessel pathology are synthesised in Table 2.

**Table 2:** Interventions targeting protein S-palmitoylation and their effects in cardiovascular

Interventions	Target	Model	Effects	References
-	Selk knockout	C57BL/6J mice	Reduced CD36 palmitoylation limits modLDL uptake and foam-cell formation, and Selk-/- immune cells likewise lessen atherosclerotic lesions.	[183]
-	Cav1 knockout	C57BL/6J mice	Cav1 knockout raises NO, S-nitrosating CD36 at palmitoylation sites, blocking its membrane trafficking, cutting lipid uptake and foam cells, and preventing myocardial lipotoxicity.	[184]
MI-773	STRING	C57BL/6J mice	Inhibiting STING palmitoylation for three weeks reduces infarct size and scarring, improves heart function, and alleviates cardiac hypertrophy	[185]
Disulfiram	Gasdermin D	C57BL/6J mice	Blocking Cys192 palmitoylation of GSDMD reduces myocardial pyroptosis and injury in AMI mice.	[156]
vaccarin	NLRP3	H9c2 cells C57BL/6 J mice	Palmitoylation of NLRP3 inactivates the inflammasome, reducing LPS-induced cardiomyocyte apoptosis, inflammation, oxidative stress, and mitochondrial dysfunction.	[175]
2-bromopalmitate	PKM2	HUVECs	Inhibition of palmitoylation promotes PKM2 tetramerization, alleviating PA-induced endothelial injury.	[176]
miR-574-5p	ZDHHC14	VSMCs	Overexpression of miR-574-5p promotes VSMC proliferation and inhibits apoptosis by suppressing the expression of the ZDHHC14 gene.	[186]
Disulfiram	Gasdermin D	C57BL/6J mice	Blocking Cys192 palmitoylation of GSDMD reduces myocardial pyroptosis and injury in AMI mice.	[156]

#### Strategies aimed at CD36

Spalmitoylation of the four cysteine residues in CD36 governs its plasmamembrane localization and the rate of long chain fatty acid uptake. Multiple studies now identify CD36 Spalmitoylation as a pathogenic driver of metabolic remodeling. In a murine model of acute myocardial infarction, AAV9mediated overexpression of a depalmitoylationdeficient CD36 mutant, as well as pharmacological inhibition of palmitoylation with SSO or 2bromopalmitate, improved ejection fraction and reduced infarct size, confirming that CD36 Spalmitoylation underlies postinfarction remodeling [12]. In vivo, deletion of the bileacid receptor TGR5 upregulates DHHC4mediated palmitoylation of CD36, enhances its membrane localisation and drives cardiac lipid loading, culminating in systolic dysfunction [174]. Complementary work shows that Selknull mice display diminished CD36 palmitoylation in bonemarrowderived macrophages, attenuated modLDL uptake and foamcell formation, and reduced atherosclerotic lesion development without impairing leukocyte homing to the arterial wall [183]. The FoxO1–zDHHC4–CD36 axis has been validated in db/db mice, diabetic pigs, and human iPSCderived cardiomyocytes. Cardiacspecific knockdown of zDHHC4 or pharmacological inhibition of its activity—markedly reduces CD36 palmitoylation, restores fatty acid metabolism, and improves contractile function [11]. Endotheliumspecific Cav1 knockout male mice develop hyperlipidaemia irrespective of diet yet maintain endothelial performance; mechanistically, loss of Cav1 elevates NO, which Snitrosates CD36 at the same cysteines that accept palmitate, competitively blocking palmitoylation and membrane targeting, thereby curbing lipid uptake, foamcell formation and myocardial lipotoxicity [184]. In sum, convergent genetic and pharmacological evidence positions CD36 palmitoylation as a tractable target for mitigating metabolic remodelling in cardiovascular disease.

#### Inflammation pathways

Beyond CD36, the Spalmitoylation of inflammationrelated proteins has also been shown to be a key regulatory node in cardiovascular injury. The STING inhibitor MI-773 hinders postinfarct adverse remodelling by suppressing STING palmitoylation and oligomerisation, which improves scar healing and slows progression to ischaemic heart failure [185]. Disulfiram antagonises palmitoylation of Cys192 on the GSDMDN terminus, thereby alleviating pyroptosisdriven myocardial injury in acute myocardial infarction [156]. In septic cardiotoxicity, vaccarin engages zDHHC12 to promote palmitoylationdependent inactivation of the NLRP3 inflammasome, mitigating LPStriggered cardiomyocyte apoptosis, inflammation, oxidative stress and mitochondrial dysfunction; the acylation inhibitor 2bromopalmitate abrogates vaccarin’s benefit, underscoring the mechanistic importance of NLRP3 palmitoylation [175]. These data together reveal that selective interference with STING, GSDMD or NLRP3 palmitoylation can blunt inflammatory or pyroptotic damage across distinct cardiac injury contexts.

#### Endothelial palmitoylation modulators

Within endothelial cells, palmitoylation of pyruvatekinase isoform M2 disrupts its tetramerisation, suppresses glycolysis and contributes to palmitateinduced endothelial damage; conversely, 2bromopalmitate rescues endothelial integrity by blocking this lipidation event [176]. MicroRNAs also enter the regulatory circuitry: serum and endothelial miR-574-5p levels are elevated in CAD, and miR-574-5p overexpression fosters vascularsmoothmusclecell proliferation while inhibiting apoptosis by silencing ZDHHC14, nominating miR-574-5p as a putative molecular target in CAD therapy [186]. Furthermore, systemic Zdhhc21^dep/dep^deficient mice display diminished microvascular barrier leakage, reduced leukocyte adhesion, and improved survival under septic or inflammatory stress. Endotheliumspecific reexpression of Zdhhc21 fully reinstates the pathological phenotype, confirming a decisive role for DHHC21dependent palmitoylation in controlling vascular permeability [152]. Likewise, siRNAmediated knockdown of DHHC21 markedly lowers eNOS palmitoylation and Golgi localization, thereby suppressing nitricoxide production and angiogenesis, whereas DHHC21 overexpression rescues these defects—highlighting a dedicated DHHC21–eNOS regulatory axis [151]. Together, these findings indicate that palmitoylationcentered, multitarget intervention strategies are steadily emerging as promising therapeutic avenues for cardiovascular disease. Altogether, modulation of endothelial palmitoylation, whether through metabolic enzymes, microRNA circuits, or DHHC21–eNOS coupling, offers a multifaceted approach to preserving vascular homeostasis.

Overall, the collective evidence spanning metabolic reprogramming, inflammatory signalling and endothelial integrity underscores the emergence of palmitoylationcentred, multitarget strategies as a promising therapeutic frontier in cardiovascular disease.

### Conclusion and future perspectives

This review has underscored the intricate crosstalk between dysregulated lipid metabolism and protein Spalmitoylation in atherosclerosis, ischaemia–reperfusion injury, cardiac arrhythmias and heart failure. Hypertriglyceridaemia, low HDLC and oxidised LDL act in concert to initiate and destabilise atherosclerotic plaques, whereas obesity, metabolic syndrome and nonalcoholic fattyliver disease magnify cardiovascular risk via chronic inflammation and lipotoxic stress. As a reversible lipid posttranslational modification, Spalmitoylation is governed by the FASNACSL metabolic axis and the availability of exogenous fatty acids; in turn, it decisively determines the membrane residency and stability of CD36, eNOS, multiple ion channels, heterotrimeric Gproteins and pyroptotic effectors. Proofofconcept studies in animal models and early pharmacological work reveal that perturbing the ZDHHC/APT enzymatic machinery—or selectively blocking aberrant palmitoylation on key substrates—can simultaneously suppress foamcell formation, enhance endothelial relaxation, stabilise cardiac electrophysiology and dampen ischaemic inflammation, thereby offering a finely tuned therapeutic avenue beyond conventional lipidlowering strategies.

Looking ahead, singlecell multiomics combined with highresolution mass spectrometry will enable construction of comprehensive “lipidome–palmitoylome” atlases across disease stages and organ systems, pinpointing regulatory nodes with the greatest translational promise. Coupled with cryoEMguided structural prediction, these datasets will clarify the substrate specificity and dynamic conformations of the ZDHHC–APT enzyme network, paving the way for highly selective smallmolecule modulators. Clinically, prospective cohort studies are warranted to quantify the incremental value of palmitoylation biomarkers in event prediction and therapeutic stratification. Systematic evaluation in largeanimal models and earlyphase trials should interrogate the safety and longterm benefit of palmitoylationtargeted agents—alone and in rational combination with lipidlowering or antiinflammatory drugs—before their potential incorporation into precision cardiovascular medicine.

## Data Availability

No datasets were generated or analysed during the current study.
